# The driver of dengue fever incidence in two high-risk areas of China: A comparative study

**DOI:** 10.1038/s41598-019-56112-8

**Published:** 2019-12-20

**Authors:** Keke Liu, Xiang Hou, Yiguan Wang, Jimin Sun, Jianpeng Xiao, Ruiyun Li, Liang Lu, Lei Xu, Shaowei Sang, Jianxiong Hu, Haixia Wu, Xiuping Song, Ning Zhao, Dongming Yan, Jing Li, Xiaobo Liu, Qiyong Liu

**Affiliations:** 1 State Key Laboratory of Infectious Disease Prevention and Control, Collaborative Innovation Center for Diagnosis and Treatment of Infectious Disease, National Institute for Communicable Disease Control and Prevention, Chinese Center for Disease Control and Prevention, Beijing, 10010 China; 2 Shandong Academy of Clinical Medicine, Shandong Provincial Hospital, Jinan, 250021 China; 3 Division of Environmental Health, School of Public Health and Management, Weifang Medical University, Weifang, 261053 Shandong Province P.R. China; 4 Shaanxi Key Laboratory for Animal Conservation, Shaanxi Institute of Zoology, Xi’an, 710032 China; 5 School of Biological Sciences, University of Queensland, Queensland, QLD 4072 Australia; 6 Zhejiang Provincial Center for Disease Control and Prevention, Hangzhou, China; 7 Guangdong Provincial Institute of Public Health, Guangdong Provincial Center for Disease Control and Prevention, Guangzhou, 511430 China; 8 MRC Centre for Global Infectious Disease Analysis, Department of Infectious Disease Epidemiology, School of Public Health, Faculty of Medicine, Imperial College London, London, W21PG United Kingdom; 9 Clinical Epidemiology Unit, Qilu Hospital of Shandong University, Jinan, 250021 China; 10 Shandong University Climate Change and Health Center, School of Public Health, Shandong University, Jinan, 250012 Shandong China

**Keywords:** Environmental impact, Viral infection

## Abstract

In China, the knowledge of the underlying causes of heterogeneous distribution pattern of dengue fever in different high-risk areas is limited. A comparative study will help us understand the influencing factors of dengue in different high-risk areas. In the study, we compared the effects of climate, mosquito density and imported cases on dengue fever in two high-risk areas using Generalized Additive Model (GAM), random forests and Structural Equation Model (SEM). GAM analysis identified a similar positive correlation between imported cases, density of Aedes larvae, climate variables and dengue fever occurrence in the studied high-risk areas of both Guangdong and Yunnan provinces. Random forests showed that the most important factors affecting dengue fever occurrence were the number of imported cases, BI and the monthly average minimum temperature in Guangdong province; whereas the imported cases, the monthly average temperature and monthly relative humidity in Yunnan province. We found the rainfall had the indirect effect on dengue fever occurrence in both areas mediated by mosquito density; while the direct effect in high-risk areas of Guangdong was dominated by temperature and no obvious effect in Yunnan province by SEM. In total, climate factors and mosquito density are the key drivers on dengue fever incidence in different high-risk areas of China. These findings could provide scientific evidence for early warning and the scientific control of dengue fever in high-risk areas.

## Introduction

Dengue fever (DF) is one of the important vector-borne viral diseases, with *Aedes albopictus* and *Ae.aegypti* as the most important vectors for viral transmission between humans^1^. It was estimated that about 500,000 people are hospitalised with severe dengue each year in the world, and the incidence of severe dengue has increased 30-fold over the past 50 years^1^. Dengue fever is endemic in many tropical and subtropical regions in the world, especially in Southeast Asia, southern Africa and the Western Pacific^2^. In mainland of China, dengue fever was a localized epidemic disease caused by imported cases with several outbreaks of dengue fever in southern provinces since 2012, especially in Guangdong^3–5^ and Yunnan provinces^6–8^. After 1949, the first large-scale outbreak of dengue fever occurred in Foshan city, Guangdong province in 1978 followed by three major outbreaks in the province in 1980, 1981 and 2014^6,9^ with more than 10,000 cases of dengue fever for every year, including more than 45,000 cases of dengue fever in 2014^10–13^. The first outbreak of dengue fever in Yunnan province occurred in 2008 since 1949, and followed by two successive outbreaks in 2013 and 2014^6,11^. Previous studies also pointed out that Guangdong and Yunnan are the major high-risk areas of dengue fever in China^14^. Currently, the knowledge of the underlying causes of heterogeneous distribution pattern of dengue fever in different high-risk areas in China is limited.

The spread process of dengue fever involved three links: the source of infection, the route of transmission and the susceptible population, which were widely affected by natural environment and socio-economic factors^2,15–18^. One study predicted that the local spatial variations of dengue risk areas was strongly influenced by temperature, rainfall and the degree of urbanisation^2^. Dengue fever only existed where the mosquito vectors, *Ae. aegypti* and *Ae. albopictus* were present, while temperature, precipitation and socio-economic factors significantly influenced the distribution of the mosquito vectors^19^. Some studies^3,4,10,20–28^ investigated the influencing factors on dengue epidemics in Guangdong province involving imported cases, mosquitoes density, temperature, precipitation, the El Niño-Southern Oscillation (ENSO), the normalized difference vegetation index (NDVI) and population, but rare studies could be found about that in Yunnan province. Presently, we remain ignorant of whether the relevant factors causing dengue fever are the same in Guangdong and Yunnan provinces, and whether the mechanisms driving dengue fever are consistent. A comparative study will help us understand the influencing factors of dengue and thus benefit the prevention and control of dengue fever in different high-risk areas using limited resources.

In this study, we collected monthly dengue cases, climate factors, larval density and imported cases in the high-risk areas of Guangdong and Yunnan Provinces from 2006 to 2017. We compared the influencing factors using Generalized Additive Model (GAM), random forest and Structural Equation Model (SEM) and found the key drivers on dengue fever incidence in different high-risk areas, which help for early warning and the scientific control of dengue fever.

## Material and Method

Ethical approval for the research was granted by Chinese Center for Disease Control and Prevention Ethics Committee (No. 201214). Our study was only involved in patients’ location and the dates of onset. There was no patients’ information used in this study, and we didn’t collect patients’ samples in our study. Therefore, the ethics committee agreed that no informed consent was needed from patients. All methods in our study were used in accordance with the relevant guidelines and regulations.

### Data collection

**Figure 1:**
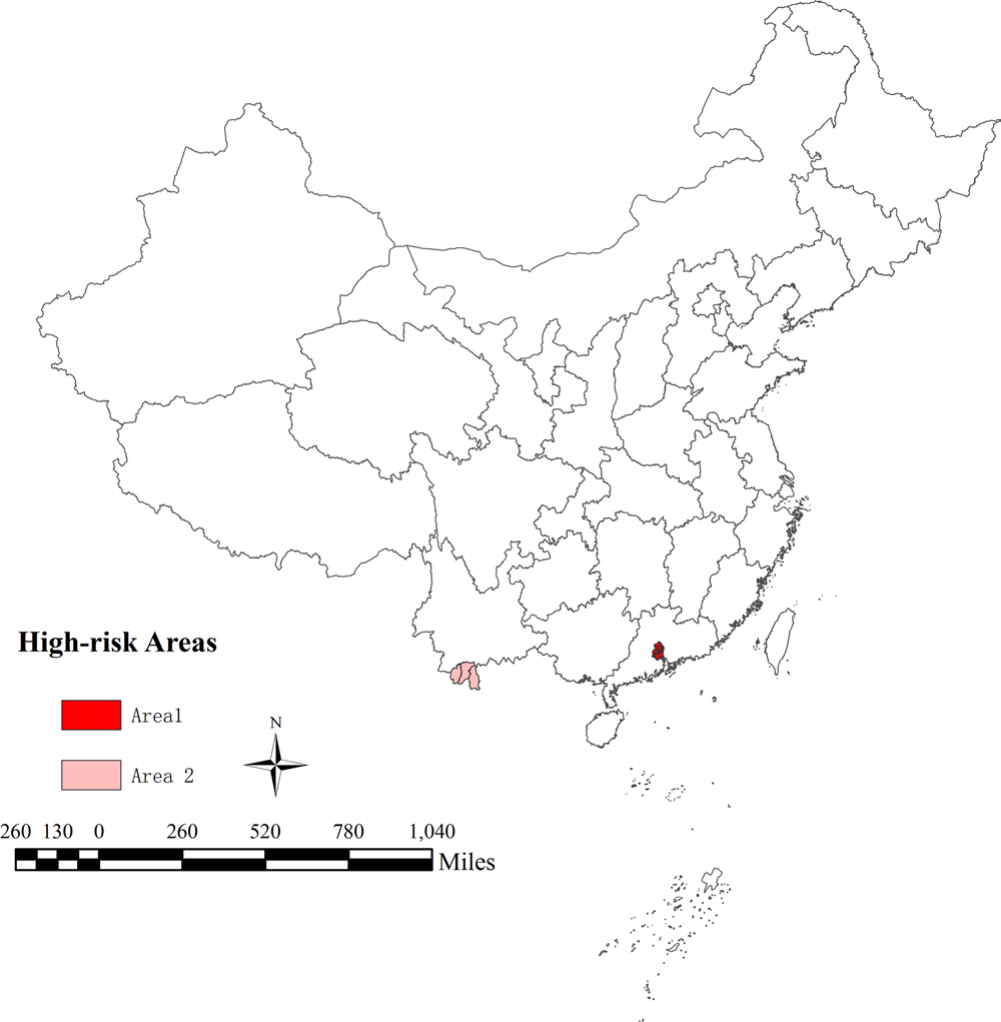
Spatial distribution of high-risk areas of Guangdong and Yunnan Provinces in China.

Two different high-risk areas in Guangdong and Yunnan provinces were selected based on the hotspot scanning and risk assessment^14^ (Fig. 1). All confirmed cases from 2006 to 2017 in the two studies areas were collected from China National Notifiable Infectious Disease Reporting Information System in Chinese Center for Disease Control and Prevention (CDC), which included national standard of current address, time of onset and diagnosis, and the source of the imported case. An imported cases of dengue fever was defined by the patients been to a dengue affected foreign country and reported being bitten by mosquitoes within 15 days of the onset of illness. Indigenous and imported cases in the two study areas were identified by surveillance system.

Meteorological data in the study areas between 2006 and 2017 were downloaded from the National Meteorological Science Data Sharing Service Platform, including monthly average temperature, the monthly average maximum temperature, the monthly average minimum temperature, average relative humidity, cumulative rainfall and the number of rainy days.

Breteau Index (BI) data from June to October in 2006–2017 were obtained from the annual surveillance report of major infectious diseases and vectors in China CDC. BI is an index for surveillance the density of *Aedes* larvae by investigating the breeding of larvae (including mosquito pupae) in small water container environment in indoor and outdoor.

The formula:

$${\rm{BI}}=\frac{{\rm{Number}}\,{\rm{of}}\,{\rm{positive}}\,{\rm{containers}}\,{\rm{for}}\,{\rm{larvae}}\,{\rm{or}}\,{\rm{pupae}}\,{\rm{of}}\,{\rm{Aedes}}\,{\rm{mosquitoes}}}{{\rm{The}}\,{\rm{number}}\,{\rm{of}}\,{\rm{inspected}}\,{\rm{houses}}\,}\times 100$$

The collected dataset of dengue indigenous cases, imported cases, the meteorological data and BI data from June to October from 2006 to 2017 were integrated for the subsequent modelling analysis.

### Statistics modelling

#### GAM analysis

The Generalized Additive Model (GAM) with a quasi-poisson distribution family (link function was identity) was used to assess the effect of imported cases, BI, temperature, precipitation, humidity on dengue prevalence. The ‘mgcv’ package in R software (version 3.3.3) was used for these analyses. The model formula given by:

$${D}_{i}={a}_{i}+b({Y}_{i}\,)+c({I}_{i-1})+d({B}_{i})+e({T}_{i})+f({P}_{i})+g({H}_{i})+{\varepsilon }_{i}$$

where ${D}_{i}$ is the log transformed dengue cases in the present month *i*. Parameter ${a}_{i}$ is the overall intercept, $b({Y}_{i})$is a smooth (natural cubic spline function with the maximally 3 degrees of freedom) function of year, $c({I}_{i-1})$ is a smooth (natural cubic spline function with the maximally 3 degrees of freedom) function of the linear of log transformed dengue imported cases (added 1 to avoid taking the logarithm of zeros) in the previous month, $d({B}_{i})$ is a smooth function (natural cubic spline function with the maximum 3 degrees of freedom) of the log(BI) (added 1 to avoid taking the logarithm of zeros) in the month *i*. $\,e({T}_{i})$ is a smooth (natural cubic spline function with the maximally 3 degrees of freedom) function of temperature in the month *i* and $f({P}_{i})$ is a smooth (natural cubic spline function with the maximally 3 degrees of freedom) function of precipitation in the month *i*. ${\varepsilon }_{i}$ are uncorrelated random errors of zero mean and finite variance. Model was selected by using the generalized cross validation value (GCV)^29^, deviance explained value and the ecological theory. Higher deviance explained value and lower GCV indicated a better fit of the model.

#### SEM analysis

SEM can estimate the structural correlation among the studied variables. In this study, we used SEM to analyse the structural linear correlation between temperature, precipitation, density of mosquito larvae and dengue incidence of two high risk areas. The effects of temperature and precipitation on dengue fever were divided into direct and indirect effects. The correlation between studied variables and dengue fever were assessed. The optimal model was determined according to the Comparative Fit Index (CFI) and the Root Mean Square Error of Approximation (RMSEA). CFI ranges from 0 to 1 and larger value indicates a better overall effect of the model. For RMSEA, the smaller value indicates a better performance of the model, eg. RMSEA < 0.1 and < 0.05 indicating the good fitting, respectively.

#### Random forest analysis

Random forest is a commonly-used machine learning algorithm to classify, discriminate and solve regression problems. It can also evaluate the relative importance of each feature variable by calculating the increase of prediction error when data for that variable is permuted while all others are left unchanged. In this study, we applied random forest to identify the importance of study variables on dengue incidence of two high risk areas. The random forest algorithm evaluates the importance of a variable and Increase in the Mean of Squared Residuals (MSE) was used to evaluate the importance of studying variables.

## Results

### Monthly distribution of dengue fever incidence and its influencing factors

**Figure 2:**
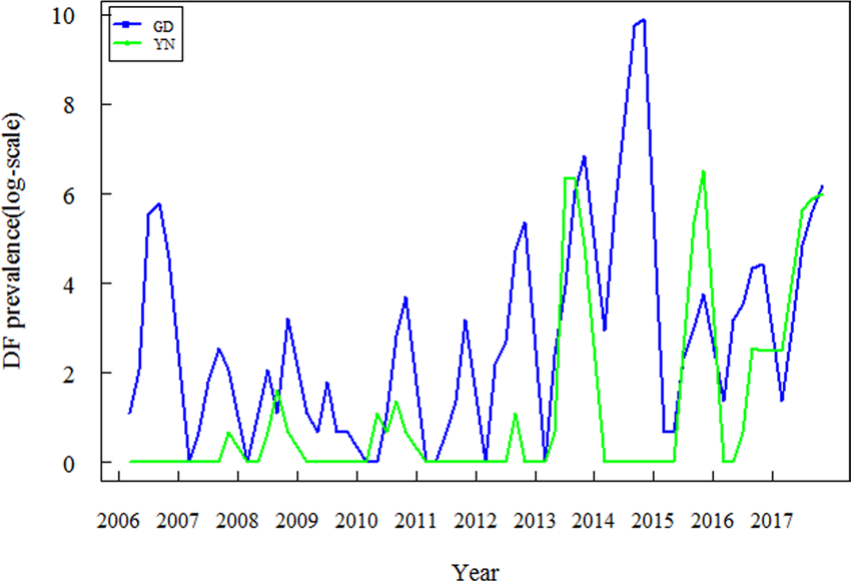
Temporal dynamics of dengue human cases in high risk areas of Guangdong and Yunnan Provinces in 2006–2017.

The temporal dynamics of dengue fever incidence in 2006–2017 was distinct in high risk areas of Guangdong and Yunnan provinces (Fig. 2). Dengue incidence in Guangdong was in general higher than that in Yunnan Province. In particular, there was an outbreak in the high-risk areas of Guangdong Province in 2014 with 38,988 indigenous cases, but no indigenous cases in high-risk areas of Yunnan Province. In 2015 and 2017, however, the number of indigenous cases in the high-risk areas of Yunnan Province was higher than that in Guangdong Province.

**Figure 3:**
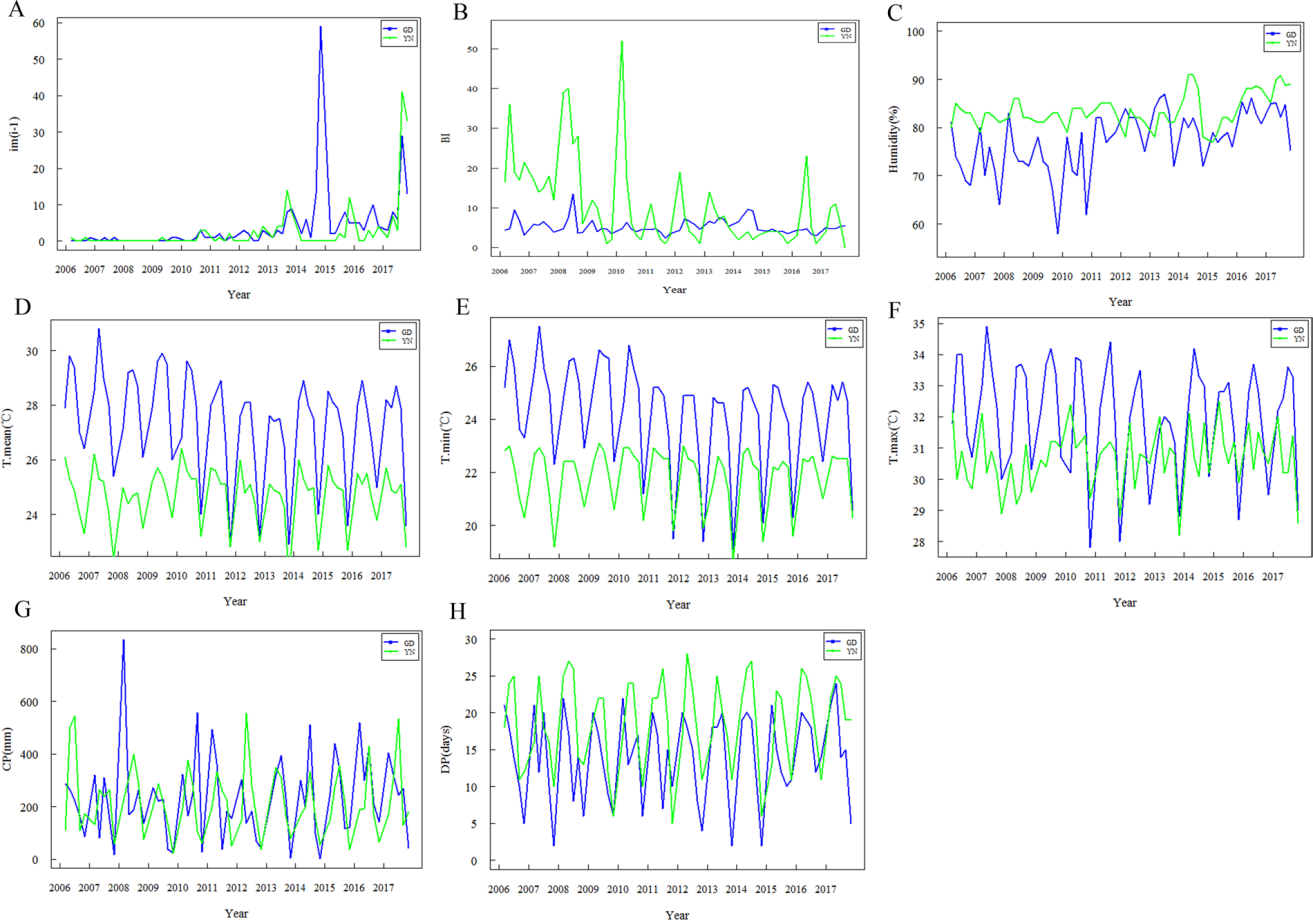
Temporal dynamics of every variable in Guangdong and Yunnan Provinces in 2006–2017. (**A**) Imported cases at last month (im(i–1)). (**B**) Breteau index (BI). (**C**) Relative humidity (Humidity). (**D**) Monthly average temperature (T-mean). (**E**) Monthly average minimum temperature (T-min). (**F**) monthly average maximum temperature (T-max). (**G**) Cumulative amount of rainfall (CP). (**J**) Monthly rainfall days (DP).

There was a significant seasonality in the temporal dynamics of influencing factors in Guangdong and Yunnan Provinces from 2006 to 2017 (Fig. 3). Compared with the meteorological conditions in Yunnan Province, Guangdong was in general of higher temperature, lower relative humidity and less rainfall days, but similar amount of rainfall. Despite the relatively more imported cases in Guangdong than Yunnan Province in 2014, there was a comparable change in other years in two different high-risk areas. BI fluctuated within a stable range in high-risk areas of Guangdong Province, but the fluctuation range in high-risk areas of Yunnan Province was larger, especially between 2006 and 2010. BI, monthly average temperature, monthly average minimum temperature, monthly average maximum temperature, monthly relative humidity, monthly cumulative rainfall and rainfall days in high-risk areas of Guangdong and Yunnan Provinces were 9.5 and 0, 1 and 0, 4.75 and 6, 27.9 and 25, 24.9 and 22.4, 32.45 and 30.8, 78.5% and 83%, 225.8 and 1999.6 mm, 15 and 19 days respectively. The minimum, maximum and quartile values of every variable were presented in the Supplementary Tables (Tables S1 and S2).

The results of correlation analysis about variables revealed high correlations among monthly average temperature (T.mean), monthly average maximum temperature (T.max) and monthly average minimum temperature (T.min). In addition, there was also a high correlation between monthly cumulative rainfall (CP) and monthly rainfall days (DP) (Tables S3 and S4**)**. Therefore, in the generalized additive model and structural equation model, three variables of temperature and two variables of rainfall were introduced into the model respectively.

### Dose-response relationship

**Figure 4:**
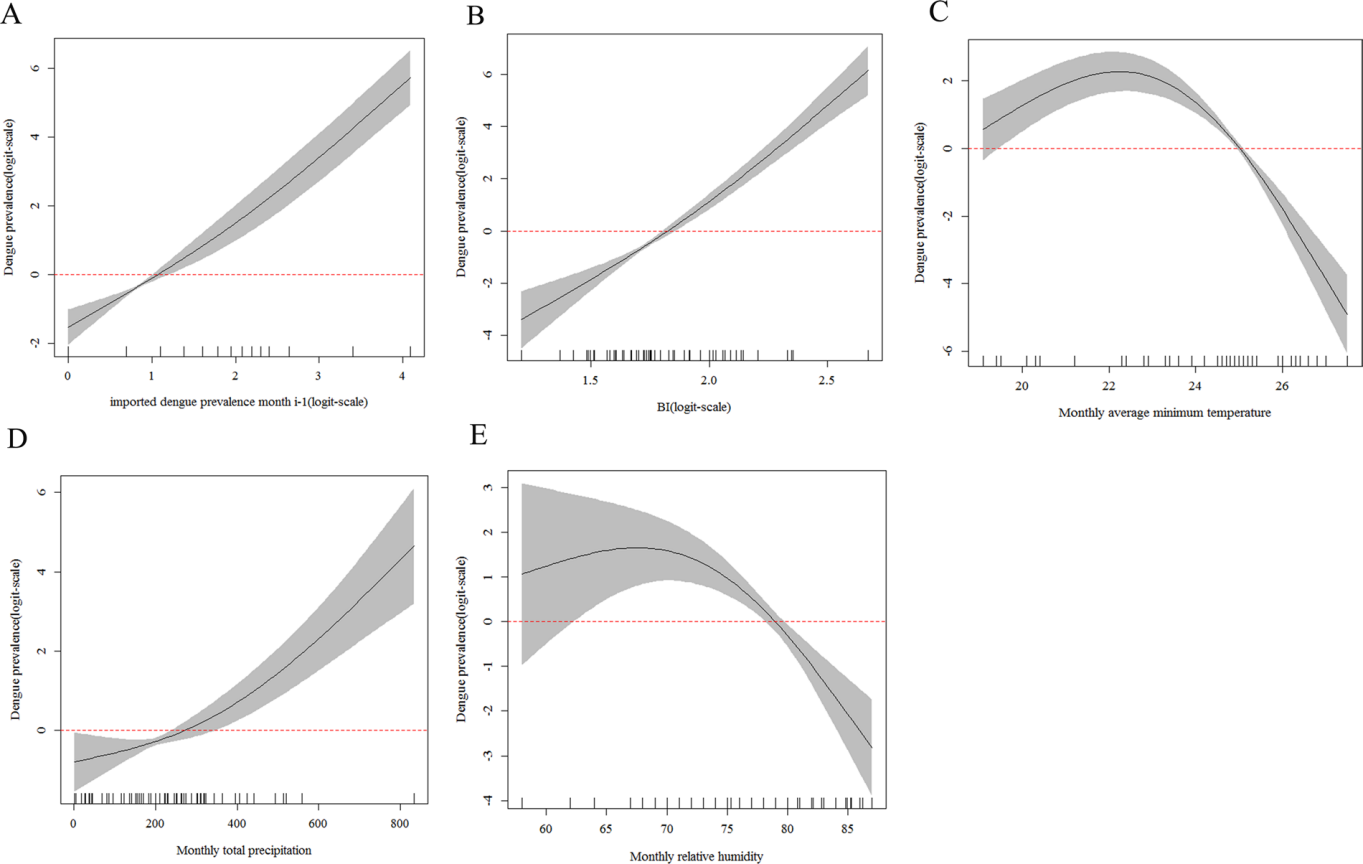
Partial effects on the dengue prevalence (log scale) based on the monthly data from 2006 to 2017 in high-risk areas of Guangdong Province. (**A**) The effect of imported cases at last month. (**B**) The effect of BI. (**C**) The effect of the monthly average minimum temperature (°C). **(D**) The effect of the monthly total precipitation (mm). (**E**) The effect of monthly relative humidity. Black lines indicate the 95% confidence intervals and red dashed line showed the threshold.

**Figure 5:**
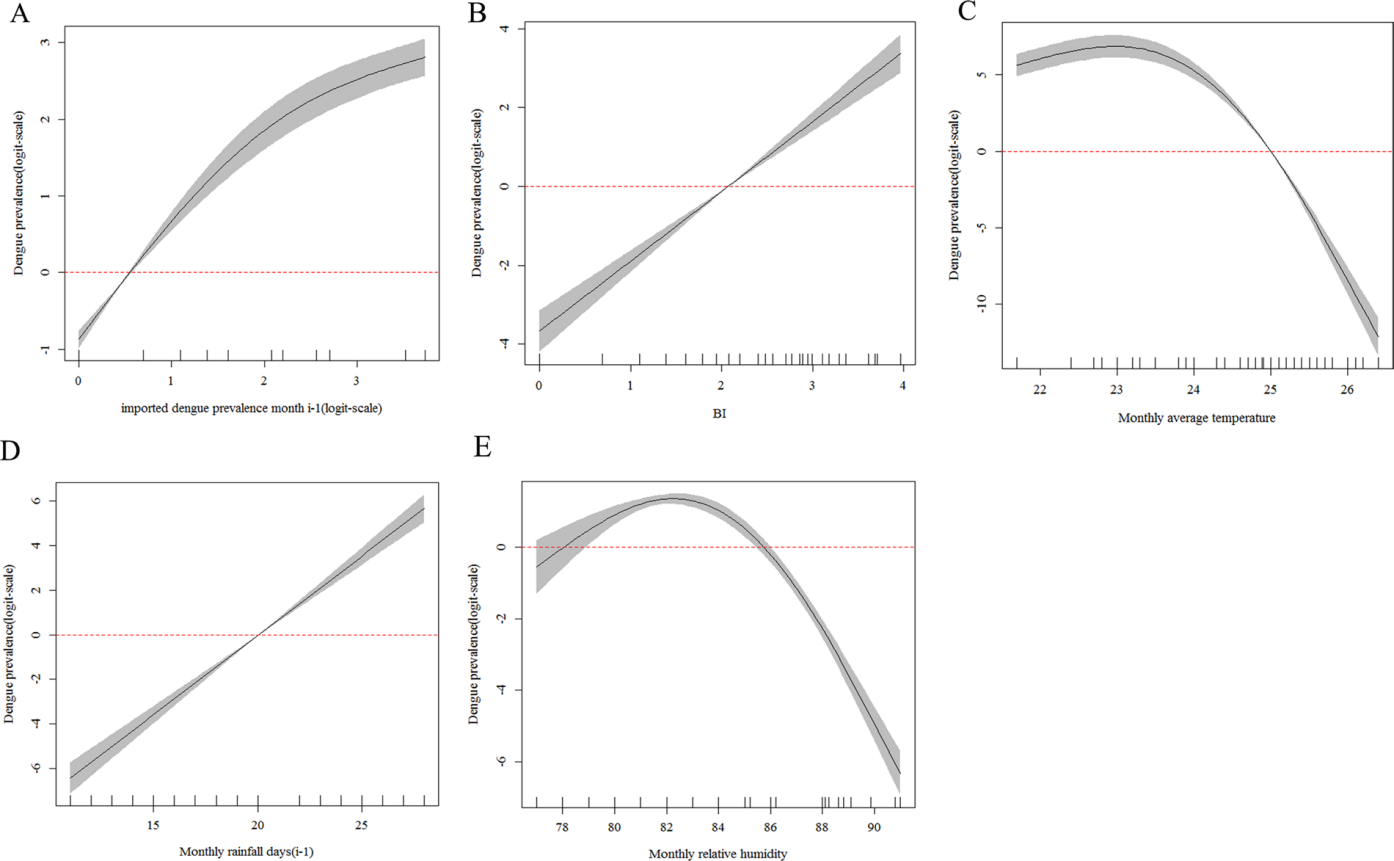
Partial effects on the dengue prevalence (log scale) based on the monthly data from 2006 to 2017 in high-risk areas of Yunnan Province. (**A**) The effect of imported cases at last month. (**B**) The effect of BI. (**C**) The effect of the monthly average temperature (°C). (**D**) The effect of the rainfall days at last month. (**E**) The effect of monthly relative humidity. Black lines indicate the 95% confidence intervals and red dashed line showed the threshold.

The influencing factors included imported cases at last month, BI, monthly average minimum temperature, monthly cumulative precipitation and monthly relative humidity about high-risk areas of Guangdong Province in the best model (Table S5). The difference was monthly average temperature and rainfall days at last month about high-risk areas of Yunnan Province in best model (Table S6). The relationship between dengue occurrence and different factors for two different areas were respectively shown in Figs. 4 and 5. GAM revealed a positive nonlinear association between imported cases at last month, BI, and cumulative precipitation and dengue occurrence in high-risk areas of Guangdong Province (*F*_*1.7, 11.4*_ = *183.38*, *p *< *0.01*, Fig. 4A; *F*_*1.4, 11.4*_ = *134.31*, *p *< *0.01*, Fig. 4B; *F*_*1.7, 11.4*_ = *16.36*, *p *< *0.01*, Fig. 4D). Moreover, A dome-shaped association between the monthly average minimum temperature with dengue occurrence, with a threshold of 22.5 °C and the optimal range was 18–25 °C (*F*_*2.0, 11.4*_ = *38.57*, *p *< *0.01*, Fig. 4C). There was a similarly dome-shaped association between the monthly humidity and dengue occurrence, with a threshold of 68% and the optimal range was 60–78% (*F*_*1.8, 11.4*_ = *14.77*, *p *< *0.01*, Fig. 4E). In high-risk areas of Yunnan Province, GAM also revealed a positive nonlinear association between imported cases at last month, BI and rainfall days at last month and dengue occurrence in Guangzhou (*F*_*1.8, 9.7*_ = *235.3*, *p *< *0.01*, Fig. 5A; *F*_*1.0, 9.7*_ = *194.2*, *p *< *0.01*, Fig. 5B; *F*_*1.0, 9.7*_ = *335.6*, *p *< *0.01*, Fig. 5D respectively). Besides, A dome-shaped association between the monthly average temperature with dengue occurrence, with a threshold of 23 °C and the optimal range was 22–25 °C (*F*_*2.0, 9.7*_ = *195.4*, *p *< *0.01*, Fig. 5C). There was a similarly dome-shaped association between the monthly humidity and dengue occurrence, with a threshold of 82% and the optimal range was 78–86% (*F*_*2.0, 9.7*_ = *222.0*, *p *< *0.01*, Fig. 5E).

### Importance of variables

**Figure 6:**
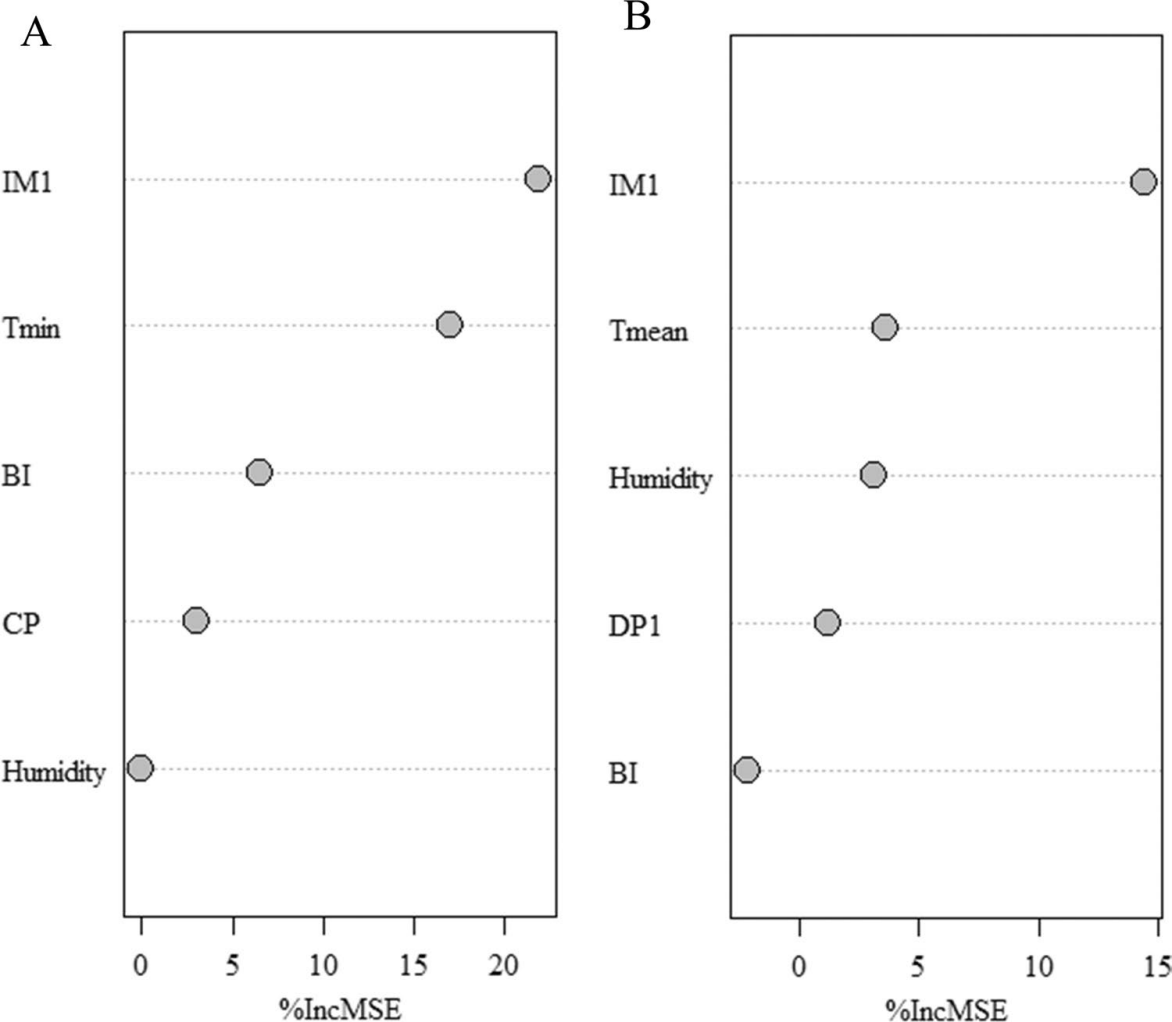
The measurement of relative importance of influencing factors on dengue fever in high risk areas of Guangdong (**A**) and Yunnan (**B**) Provinces. The importance is evaluated by using %IncMSE, increase in the mean of squared residuals (MSE).

The results of random forest showed that the most important factors affecting the occurrence of dengue fever in Guangdong high-risk areas were imported cases last month, the minimum monthly average temperature and BI, while the most important factors affecting the occurrence of dengue fever in high-risk areas of Yunnan were imported cases last month, the monthly average temperature and monthly relative humidity (Fig. 6).

### The direct and indirect effect of influencing factors on dengue incidence

**Figure 7:**
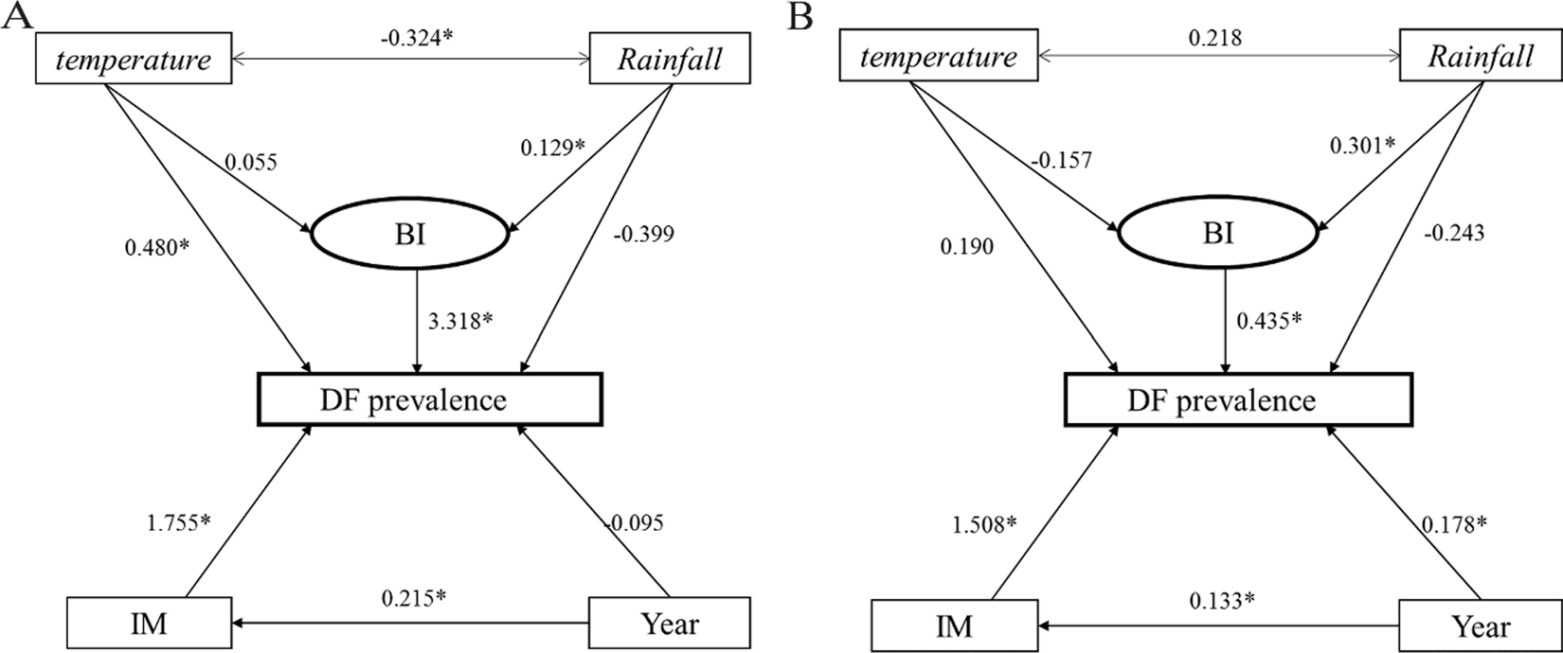
SEM analysis of the direct and indirect effect on dengue incidence in high risk areas of Guangdong (**A**) and Yunnan (**B**) Provinces. Asterisks indicated statistically significant pathways (*P* < 0.05).

We explored the direct and indirect effects of influencing factors on dengue occurrence from 2006 to 2017 using a structural equation model (SEM). The SEM results showed significant direct effect of temperature and indirect effect of precipitation on dengue incidence in high-risk areas of Guangdong Province (CFI = 0.968, RMESA = 0.104); However, the result suggested the only indirect effect of precipitation on dengue incidence in high risk areas of Yunnan Province (CFI = 0.773, RMESA = 0.261) (Fig. 7).

## Discussion

Dengue fever is one of the most important vector-borne infectious disease which is caused by four serotypes of dengue virus, mainly transmitted through the bites of mosquitoes^30^. Dengue fever has haunted in China for more than 40 years and particularly prevalent in Guangdong and Yunnan provinces with several severe outbreaks^6,9,11^. Although many studies^4,21,22,25,31,32^ were trying to investigate the influencing factors on the transmission of dengue fever in mainland of China, different conclusions were drawn partly due to the heterogeneity of time and space. For example, Cheng, Q. *et al*.^27^ found that the most important factor determining dengue outbreak pattern in 2014 in Guangzhou was an early imported case, and some meteorological factors only acted to change the transmission dynamics in the outbreak. Subsequently, they^28^ raised in 2017 that the higher number of imported cases in May and June was responsible for the early outbreak instead of climate. Oidtman, R. J. *et al*.^33^ in 2019 indicated that while dengue epidemic in most years in Guangzhou were limited by one or more unfavorable conditions such as imported cases, mosquito density and temperature, the dengue outbreak in 2014 was made possible by the combination of many favorable conditions. In addition, most studies^4,10,25,34,35^ about dengue fever focused on Guangdong Province while dengue studies in Yunnan province remained scarce. We were not clear whether the mechanism driving dengue fever was consistent in Guangdong and Yunnan provinces, which brought us lots of confusion in decision making to prevent and control of dengue fever in different high-risk areas. In this study, the climate, biological and imported cases affecting the incidence of dengue fever were, for the first time, systematically analysed in the same time range but two different areas, guiding the implementation of targeted prevention and control of dengue fever in different high-risk areas.

The findings suggested that the number of imported cases and the density of *Aedes* larva were positively correlated with the occurrence of dengue fever. Similar effects were found in many other studies^4,10^ in different regions. For example, a long-term study found that dengue fever was still an imported disease in Guangzhou, with Southeast Asian countries as major transmission sources^34^.Cheng *et al*.^28^ also found that the higher number of imported cases in May and June likely to be cause of dengue outbreak in Guangzhou, 2014. Guangdong province is located in the coastal area of China, with a large number of floating population. Likewise, Yunnan province borders with three Southeast Asian countries where dengue is endemic. The special locations of two provinces provides a convenient way for imported cases of dengue fever into China, resulting in an increase in the number of local cases of dengue fever. Besides, the subtropical monsoon climate and local weather conditions provide ideal environment for the reproduction of mosquito, which spread virus among humans through biting patients, leading to the increased incidence of dengue fever^35^.

Temperature and rainfall play a role in the different dengue fever incidence areas in Guangdong and Yunnan Provinces, affecting the different incidence of dengue fever in the two regions. Despite the similar association between dengue outbreak risk and temperature, rainfall and relative humidity, the impact of meteorological conditions on dengue outbreaks differed in two provinces. Specifically, consistent with previous studies^10,26^, monthly minimum temperature and cumulative amount of rainfall were the optimal temperature and rainfall index in GAM of high-risk areas of Guangdong Province. However, monthly average temperature and the number of rainy days in the last month were the optimal temperature index in Yunnan Province. Arcari *et al*. presented a study of the relationship between dengue fever and meteorological factors in eight Indonesian cities, which argued that rainfall drives the heterogeneity of dengue fever in different regions; whereas temperature was more likely to shape the intensity of dengue fever in a region^36^. In general, although the drivers are slightly different in the study, they still are probably driven by the same biological mechanisms about rainfall and temperature.

Random forests showed that the importance of influencing factors on dengue incidence of different high-risk areas was not identical. The imported cases in last month, the monthly average minimum temperature and BI was found to be the most important factors in the high-risk areas of Guangdong province. However, the imported cases, monthly average temperature and monthly relative humidity were the most important factors in Yunnan province. The imported cases were of great significance to the occurrence of dengue fever and the suitable temperature were conducive to the reproduction of *Aedes* mosquitoes and dengue virus replication^4,37,38^. In addition, we speculated that the vector density index selected in different areas should be different as BI was found more important in Guangdong than Yunnan province. However, In the study of different regions, different conclusions were drawn between BI and dengue fever occurrence^39–41^. For example, A meta-analysis involving 18 items on the relationship between mosquito vector index and dengue fever showed that there was no inevitability between the number of cases of dengue fever and the mosquito vector index^41^. Therefore, it is necessary to further explore appropriate evaluation index of mosquito density in different regions.

In the structural equation model, we also found different paths through which climatic factors affect dengue fever in different regions. The appropriate temperature in different areas has different effect on dengue fever. The direct effect of temperature on dengue fever was significant in Guangdong, but not significant in Yunnan. Rainfall had indirect effect on dengue fever occurrence in both studied areas. Rainfall can provide more breeding environment for *Aedes* mosquito larvae and thus affect the occurrence of dengue fever^3,42^. Similarly, we found that the rainfall and relative humidity in Yunnan were generally higher than that of Guangdong, which may also be important indicators to distinguish meteorological conditions and their impact on dengue outbreak risks in Guangdong and Yunnan Provinces.

Some limitations should be considered when interpreting our findings. Firstly, the bias could be existed in our analysis because the dengue cases came from the passive surveillance system called the China National Notifiable Infectious Disease Reporting Information System. Secondly, besides meteorological factors, imported cases and mosquito density, many other factors such as population immunity, population awareness, social factors and urbanization level may also have effects on dengue outbreaks but were not included in the analysis due to data unavailable. Thirdly, by assuming a homogeneous distribution of larvae density across Xishuangbanna Dai Autonomous Prefecture of Yunnan Province, density index of *Aedes* larvae in the county JingHong is used as the proxy of larvae density of the entire Xishuangbanna Dai Autonomous Prefecture. This assumption is made sue partly to the sampling bias as the density data is only available in the county JingHong. It may lead to the underestimation of the heterogeneity of larvae density and hence the outbreak risks in high-risk areas of Yunnan Province.

In summary, our study suggested that climate factors and mosquito density are the key drivers on dengue incidence in different high-risk areas dengue fever incidence. These findings provide scientific evidence for early warning systems to help to guide the allocation of limited resources to targeted areas and controls of dengue fever.

## Supplementary information

Supplementary Materials

## Data Availability

All data involved in the study are available from Qiyong Liu and Xiaobo Liu.
