# Supplementary material for: The driver of dengue fever incidence in two high-risk areas of China: A comparative study
Source: Sci Rep. 2019 Dec 20;9:19510. doi: 10.1038/s41598-019-56112-8 (PMC6925307; doi:10.1038/s41598-019-56112-8)
Supplement: Supplementary file 1 — Supplementary Materials [file 41598_2019_56112_MOESM1_ESM.docx]

Supplementary Materials for

**Title: The driver of dengue fever incidence in two high-risk areas of China: A comparative study**

Author: Keke Liu^1,2,3^, Xiang Hou^4^, Yiguan Wang^5^, Jimin Sun^6^, Jianpeng Xiao^7^, Ruiyun Li^8^, Liang Lu^1^, Lei Xu^1^, Shaowei Sang^9^, Jianxiong Hu^7^, Haixia Wu^1^, Xiuping Song^1^, Ning Zhao^1^, Dongming Yan^1^, Jing Li^3,*^, Xiaobo Liu^1,^^*^, Qiyong Liu^1,10*^

*corresponding authors: [liuqiyong@icdc.cn](mailto:liuqiyong@icdc.cn) and [liuxiaobo@icdc.cn](mailto:liuxiaobo@icdc.cn) and lijingsddx@126.com

|  | Cases | IM | BI | T_mean | T_min | T_max | Hum | CP | DP |
| --- | --- | --- | --- | --- | --- | --- | --- | --- | --- |
| Min. | 0.00 | 0.00 | 2.35 | 22.80 | 19.10 | 27.80 | 58.00 | 1.20 | 2.00 |
| 1st Qu. | 1.75 | 0.00 | 4.15 | 26.70 | 23.55 | 31.27 | 72.75 | 133.1 | 10.00 |
| Median | 9.50 | 1.00 | 4.75 | 27.90 | 24.90 | 32.45 | 78.50 | 225.8 | 15.00 |
| Mean | 713.30 | 3.95 | 5.33 | 27.45 | 24.34 | 32.11 | 77.26 | 233.6 | 14.03 |
| 3rd Qu. | 77.75 | 4.25 | 6.42 | 28.70 | 25.32 | 33.42 | 82.00 | 310.1 | 19.00 |
| Max. | 19790.0 | 59.0 | 13.46 | 30.80 | 27.50 | 34.90 | 87.00 | 834.6 | 24.00 |

**Table S1** Dengue cases and research variables in high-risk areas of Guangdong Province.

IM: imported cases at last month. BI: Breteau index. T_mean: monthly average temperature. T_min: monthly average minimum temperature. T_max: monthly average maximum temperature. Hum: monthly relative humidity. CP: monthly cumulative amount of rainfall. DP: monthly rainfall days.

**Table S2** Dengue cases and research variables in high-risk areas of Yunnan Province.

|  | Cases | IM | BI | T.mean | T.min | T.max | Hum | CP | DP |
| --- | --- | --- | --- | --- | --- | --- | --- | --- | --- |
| Min. | 0.00 | 0.00 | 0.00 | 21.70 | 18.70 | 28.20 | 77.00 | 23.60 | 5.00 |
| 1st Qu. | 0.00 | 0.00 | 3.00 | 24.38 | 21.52 | 30.20 | 81.75 | 135.30 | 15.50 |
| Median | 0.00 | 0.00 | 6.00 | 25.00 | 22.40 | 30.8 | 83.00 | 199.60 | 19.00 |
| Mean | 55.57 | 2.65 | 10.66 | 24.73 | 21.91 | 30.68 | 83.62 | 222.40 | 18.75 |
| 3rd Qu. | 2.25 | 2.25 | 15.35 | 25.30 | 22.60 | 31.20 | 85.40 | 288.30 | 24.00 |
| Max. | 672.00 | 41.00 | 52.00 | 26.40 | 23.10 | 32.50 | 91.00 | 555.90 | 28.00 |

IM: imported cases at last month. BI: Breteau index. T_mean: monthly average temperature. T_min: monthly average minimum temperature. T_max: monthly average maximum temperature. Hum: monthly relative humidity. CP: monthly cumulative amount of rainfall. DP: monthly rainfall days.

**Table S3** Analysis of correlation analysis variables in high-risk areas of Guangdong Province.

| Variables | T.mean | T.min | T.max | Hum | CP | DP |
| --- | --- | --- | --- | --- | --- | --- |
| T.mean | 1 |  |  |  |  |  |
| T.min | 0.98 | 1 |  |  |  |  |
| T.max | 0.95 | 0.89 | 1 |  |  |  |
| Hum | 0.13 | 0.18 | 0.18 | 1 |  |  |
| CP | 0.36 | 0.44 | 0.28 | 0.59 | 1 |  |
| DP | 0.55 | 0.62 | 0.46 | 0.66 | 0.75 | 1 |

T_mean: monthly average temperature. T_min: monthly average minimum temperature. T_max: monthly average maximum temperature. Hum: monthly relative humidity. CP: monthly cumulative amount of rainfall. DP: monthly rainfall days.

| Variables | T.mean | T.min | T.max | Hum | CP | DP |
| --- | --- | --- | --- | --- | --- | --- |
| T.mean | 1 |  |  |  |  |  |
| T.min | 0.95 | 1 |  |  |  |  |
| T.max | 0.78 | 0.6 | 1 |  |  |  |
| Hum | 0.05 | 0.23 | -0.18 | 1 |  |  |
| CP | 0.41 | 0.57 | 0 | 0.31 | 1 |  |
| DP | 0.61 | 0.77 | 0.15 | 0.45 | 0.75 | 1 |

**Table S4** Analysis of correlation analysis variables in high-risk areas of Yunnan Province.

T_mean: monthly average temperature. T_min: monthly average minimum temperature. T_max: monthly average maximum temperature. Hum: monthly relative humidity. CP: monthly cumulative amount of rainfall. DP: monthly rainfall days.

Table S5 The five optimal models selection results in Guangdong Province

| Model Selection | GCV | Deviance  Explained（%） |
| --- | --- | --- |
| $\boldsymbol{D}_{\boldsymbol{i}}\boldsymbol{=}\boldsymbol{a}_{\boldsymbol{i}}\boldsymbol{+b}\left( \boldsymbol{Y}_{\boldsymbol{i}} \right)\boldsymbol{+}\boldsymbol{c(}\boldsymbol{I}_{\boldsymbol{i-1}}\boldsymbol{)+d(}\boldsymbol{B}_{\boldsymbol{i}}\boldsymbol{)+e(}\boldsymbol{T}_{\boldsymbol{(min)}\boldsymbol{i}}\boldsymbol{)+f(}\boldsymbol{P}_{\boldsymbol{(CP)i}}\boldsymbol{)+g(}\boldsymbol{H}_{\boldsymbol{i}}\boldsymbol{)+}\boldsymbol{\varepsilon}_{\boldsymbol{i}}$* | 129.84 | 97.8 |
| $\boldsymbol{D}_{\boldsymbol{i}}\boldsymbol{=}\boldsymbol{a}_{\boldsymbol{i}}\boldsymbol{+b}\left( \boldsymbol{Y}_{\boldsymbol{i}} \right)\boldsymbol{+}\boldsymbol{c(}\boldsymbol{I}_{\boldsymbol{i-1}}\boldsymbol{)+d(}\boldsymbol{B}_{\boldsymbol{i}}\boldsymbol{)+e(}\boldsymbol{T}_{\boldsymbol{(min)}\boldsymbol{i}}\boldsymbol{)+f(}\boldsymbol{P}_{\boldsymbol{(DP)i-1}}\boldsymbol{)+g(}\boldsymbol{H}_{\boldsymbol{i}}\boldsymbol{)+}\boldsymbol{\varepsilon}_{\boldsymbol{i}}$ | 137.69 | 97.6 |
| $\boldsymbol{D}_{\boldsymbol{i}}\boldsymbol{=}\boldsymbol{a}_{\boldsymbol{i}}\boldsymbol{+b}\left( \boldsymbol{Y}_{\boldsymbol{i}} \right)\boldsymbol{+}\boldsymbol{c(}\boldsymbol{I}_{\boldsymbol{i-1}}\boldsymbol{)+d(}\boldsymbol{B}_{\boldsymbol{i}}\boldsymbol{)+e(}\boldsymbol{T}_{\boldsymbol{(mean)}\boldsymbol{i}}\boldsymbol{)+f(}\boldsymbol{P}_{\boldsymbol{(CP)i}}\boldsymbol{)+g(}\boldsymbol{H}_{\boldsymbol{i}}\boldsymbol{)+}\boldsymbol{\varepsilon}_{\boldsymbol{i}}$ | 145.81 | 97.5 |
| $\boldsymbol{D}_{\boldsymbol{i}}\boldsymbol{=}\boldsymbol{a}_{\boldsymbol{i}}\boldsymbol{+b}\left( \boldsymbol{Y}_{\boldsymbol{i}} \right)\boldsymbol{+}\boldsymbol{c(}\boldsymbol{I}_{\boldsymbol{i-1}}\boldsymbol{)+d(}\boldsymbol{B}_{\boldsymbol{i}}\boldsymbol{)+e(}\boldsymbol{T}_{\boldsymbol{(mean)}\boldsymbol{i}}\boldsymbol{)+f(}\boldsymbol{P}_{\boldsymbol{(DP)i-1}}\boldsymbol{)+g(}\boldsymbol{H}_{\boldsymbol{i}}\boldsymbol{)+}\boldsymbol{\varepsilon}_{\boldsymbol{i}}$ | 158.74 | 97.3 |
| $\boldsymbol{D}_{\boldsymbol{i}}\boldsymbol{=}\boldsymbol{a}_{\boldsymbol{i}}\boldsymbol{+b}\left( \boldsymbol{Y}_{\boldsymbol{i}} \right)\boldsymbol{+}\boldsymbol{c(}\boldsymbol{I}_{\boldsymbol{i-1}}\boldsymbol{)+d(}\boldsymbol{B}_{\boldsymbol{i}}\boldsymbol{)+e(}\boldsymbol{T}_{\boldsymbol{(mean)}\boldsymbol{i}}\boldsymbol{)+f(}\boldsymbol{P}_{\left( \boldsymbol{CP} \right)\boldsymbol{i-1}}\boldsymbol{)+g(}\boldsymbol{H}_{\boldsymbol{i}}\boldsymbol{)+}\boldsymbol{\varepsilon}_{\boldsymbol{i}}$ | 164.08 | 97.1 |
| $\boldsymbol{D}_{\boldsymbol{i}}\boldsymbol{=}\boldsymbol{a}_{\boldsymbol{i}}\boldsymbol{+b}\left( \boldsymbol{Y}_{\boldsymbol{i}} \right)\boldsymbol{+}\boldsymbol{c(}\boldsymbol{I}_{\boldsymbol{i-1}}\boldsymbol{)+d(}\boldsymbol{B}_{\boldsymbol{i}}\boldsymbol{)+e(}\boldsymbol{T}_{\boldsymbol{(max)}\boldsymbol{i}}\boldsymbol{)+f(}\boldsymbol{P}_{\boldsymbol{(CP)i}}\boldsymbol{)+g(}\boldsymbol{H}_{\boldsymbol{i}}\boldsymbol{)+}\boldsymbol{\varepsilon}_{\boldsymbol{i}}$ | 187.44 | 96.6 |

* The best model

Table S6 The five optimal models selection results in Yunnan Province

| Model Selection | GCV | Deviance  Explained（%） |
| --- | --- | --- |
| $\boldsymbol{D}_{\boldsymbol{i}}\boldsymbol{=}\boldsymbol{a}_{\boldsymbol{i}}\boldsymbol{+b}\left( \boldsymbol{Y}_{\boldsymbol{i}} \right)\boldsymbol{+}\boldsymbol{c(}\boldsymbol{I}_{\boldsymbol{i-1}}\boldsymbol{)+d(}\boldsymbol{B}_{\boldsymbol{i}}\boldsymbol{)+e(}\boldsymbol{T}_{\boldsymbol{(max)}\boldsymbol{i}}\boldsymbol{)+f(}\boldsymbol{P}_{\left( \boldsymbol{CP} \right)\boldsymbol{i-1}}\boldsymbol{)+g(}\boldsymbol{H}_{\boldsymbol{i}}\boldsymbol{)+}\boldsymbol{\varepsilon}_{\boldsymbol{i}}$ | 16.516 | 95.0 |
| $\boldsymbol{D}_{\boldsymbol{i}}\boldsymbol{=}\boldsymbol{a}_{\boldsymbol{i}}\boldsymbol{+b}\left( \boldsymbol{Y}_{\boldsymbol{i}} \right)\boldsymbol{+}\boldsymbol{c(}\boldsymbol{I}_{\boldsymbol{i-1}}\boldsymbol{)+d(}\boldsymbol{B}_{\boldsymbol{i}}\boldsymbol{)+e(}\boldsymbol{T}_{\left( \boldsymbol{max} \right)\boldsymbol{i-1}}\boldsymbol{)+f(}\boldsymbol{P}_{\left( \boldsymbol{CP} \right)\boldsymbol{i-1}}\boldsymbol{)+g(}\boldsymbol{H}_{\boldsymbol{i}}\boldsymbol{)+}\boldsymbol{\varepsilon}_{\boldsymbol{i}}$ | 17.337 | 94.6 |
| $\boldsymbol{D}_{\boldsymbol{i}}\boldsymbol{=}\boldsymbol{a}_{\boldsymbol{i}}\boldsymbol{+b}\left( \boldsymbol{Y}_{\boldsymbol{i}} \right)\boldsymbol{+}\boldsymbol{c(}\boldsymbol{I}_{\boldsymbol{i-1}}\boldsymbol{)+d(}\boldsymbol{B}_{\boldsymbol{i}}\boldsymbol{)+e(}\boldsymbol{T}_{\boldsymbol{(mean)}\boldsymbol{i}}\boldsymbol{)+f(}\boldsymbol{P}_{\left( \boldsymbol{CP} \right)\boldsymbol{i-1}}\boldsymbol{)+g(}\boldsymbol{H}_{\boldsymbol{i}}\boldsymbol{)+}\boldsymbol{\varepsilon}_{\boldsymbol{i}}$ | 22.685 | 92.5 |
| $\boldsymbol{D}_{\boldsymbol{i}}\boldsymbol{=}\boldsymbol{a}_{\boldsymbol{i}}\boldsymbol{+b}\left( \boldsymbol{Y}_{\boldsymbol{i}} \right)\boldsymbol{+}\boldsymbol{c(}\boldsymbol{I}_{\boldsymbol{i-1}}\boldsymbol{)+d(}\boldsymbol{B}_{\boldsymbol{i}}\boldsymbol{)+e(}\boldsymbol{T}_{\boldsymbol{(min)}\boldsymbol{i}}\boldsymbol{)+f(}\boldsymbol{P}_{\left( \boldsymbol{CP} \right)\boldsymbol{i-1}}\boldsymbol{)+g(}\boldsymbol{H}_{\boldsymbol{i}}\boldsymbol{)+}\boldsymbol{\varepsilon}_{\boldsymbol{i}}$ | 23.919 | 92.6 |
| $\boldsymbol{D}_{\boldsymbol{i}}\boldsymbol{=}\boldsymbol{a}_{\boldsymbol{i}}\boldsymbol{+b}\left( \boldsymbol{Y}_{\boldsymbol{i}} \right)\boldsymbol{+}\boldsymbol{c(}\boldsymbol{I}_{\boldsymbol{i-1}}\boldsymbol{)+d(}\boldsymbol{B}_{\boldsymbol{i}}\boldsymbol{)+e(}\boldsymbol{T}_{\boldsymbol{(max)}\boldsymbol{i}}\boldsymbol{)+f(}\boldsymbol{P}_{\boldsymbol{(DP)i}}\boldsymbol{)+g(}\boldsymbol{H}_{\boldsymbol{i}}\boldsymbol{)+}\boldsymbol{\varepsilon}_{\boldsymbol{i}}$ | 26.556 | 92.2 |
| $\boldsymbol{D}_{\boldsymbol{i}}\boldsymbol{=}\boldsymbol{a}_{\boldsymbol{i}}\boldsymbol{+b}\left( \boldsymbol{Y}_{\boldsymbol{i}} \right)\boldsymbol{+}\boldsymbol{c}\left( \boldsymbol{I}_{\boldsymbol{i-1}} \right)\boldsymbol{+d}\left( \boldsymbol{B}_{\boldsymbol{i}} \right)\boldsymbol{+e}\left( \boldsymbol{T}_{\left( \boldsymbol{mean} \right)\boldsymbol{i}} \right)\boldsymbol{+f}\left( \boldsymbol{P}_{\left( \boldsymbol{DP} \right)\boldsymbol{i-1}} \right)\boldsymbol{+g}\left( \boldsymbol{H}_{\boldsymbol{i}} \right)\boldsymbol{+}\boldsymbol{\varepsilon}_{\boldsymbol{i}}$* | 27.376 | 91.2 |

* The best model.
